# Lian-Cang Xu: the founder of management psychology in China

**DOI:** 10.1007/s13238-017-0419-1

**Published:** 2017-05-12

**Authors:** Jing Li, Yong-Kang Lu

**Affiliations:** School of Psychology, Nanjing Normal University, Nanjing, 210097 China

Dr. Lian-Cang Xu (徐联仓, 1927–2015), born in Haining County, Zhejiang Province, China, is a well-known Chinese psychologist (Fig. 1), the founder of management psychology in China, and also the pioneer of industrial psychology and engineering psychology.

**Figure 1:**
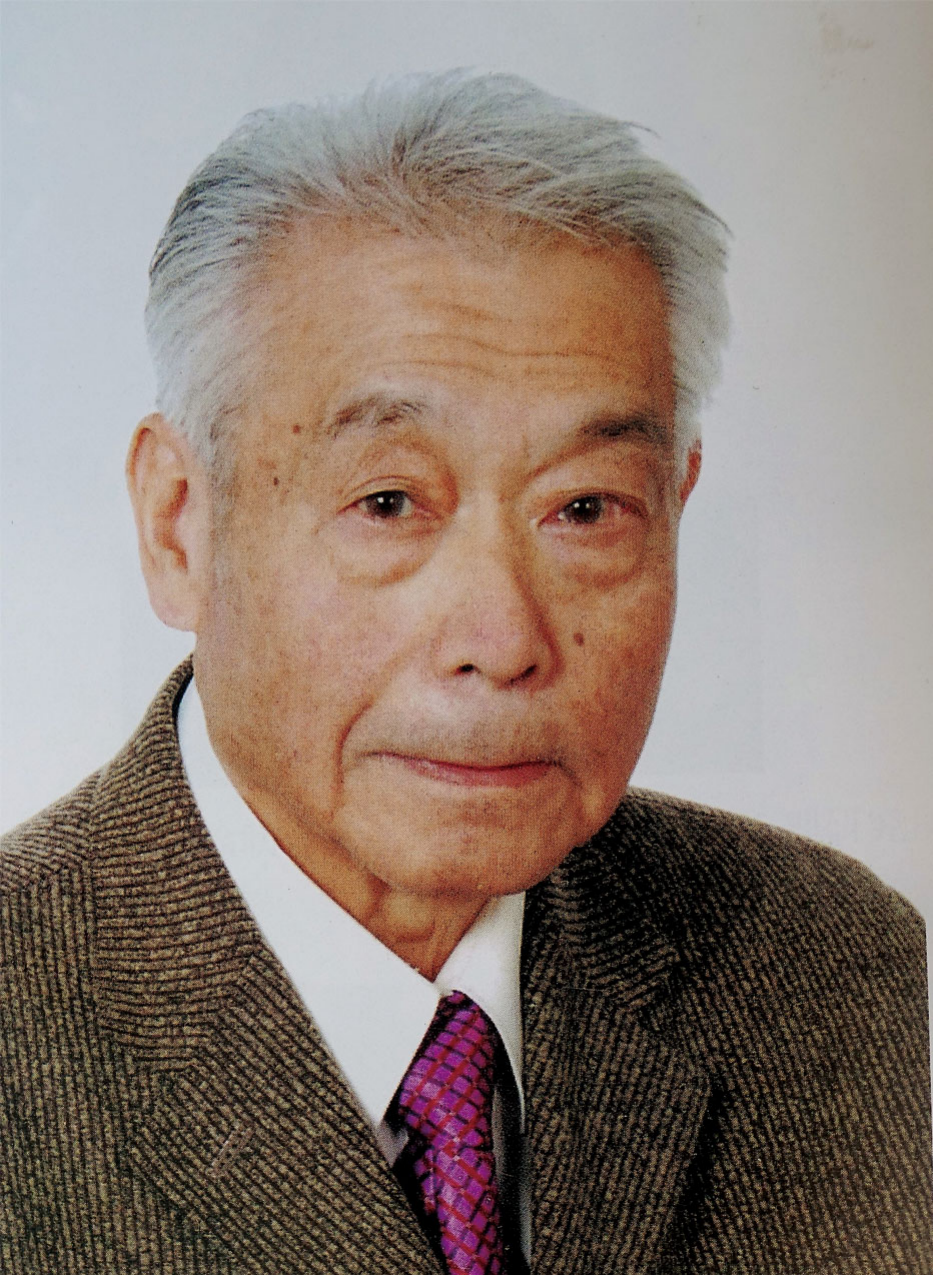
**Professor Lian-Cang Xu**.

From 1947 to 1949, Lian-Cang Xu studied in Department of Philosophical Education^1^ in Nankai University, and then transferred to Tsinghua University, majoring in psychology. After acquiring bachelor’s degree in 1951, he began his academic career in the Institute of Psychology, Chinese Academy of Sciences (IPCAS), where he worked all his life. He was mainly engaged in industrial psychology studies about quality control, production safety, and operation rationalization in the early 1950s. What’s more, these research finding were successfully popularized in Chinese textile and metallurgy industry (Fu, 2015).

Aiming to continue his education, Lian-Cang Xu went to Psychological Research Institute of Russian Soviet Federated Socialist Republic (RSFSR) Pedagogical Science Academy^2^ in 1958 (Fig. 2), and received a Kandidat Nauk degree^3^ in 1962. During this period in USSR, he introduced information theory into psychological science creatively, and proposed a brand-new analytic method of scrap in production. His theory and method were applied in a television factory experimentally and that resulted in the improvement of product quality. This study was included in a textbook named “Labor Psychology” published by Pedagogical Science Academy later. Because of these outstanding performances, he was awarded with Ulsenski Prize by the academy.

**Figure 2:**
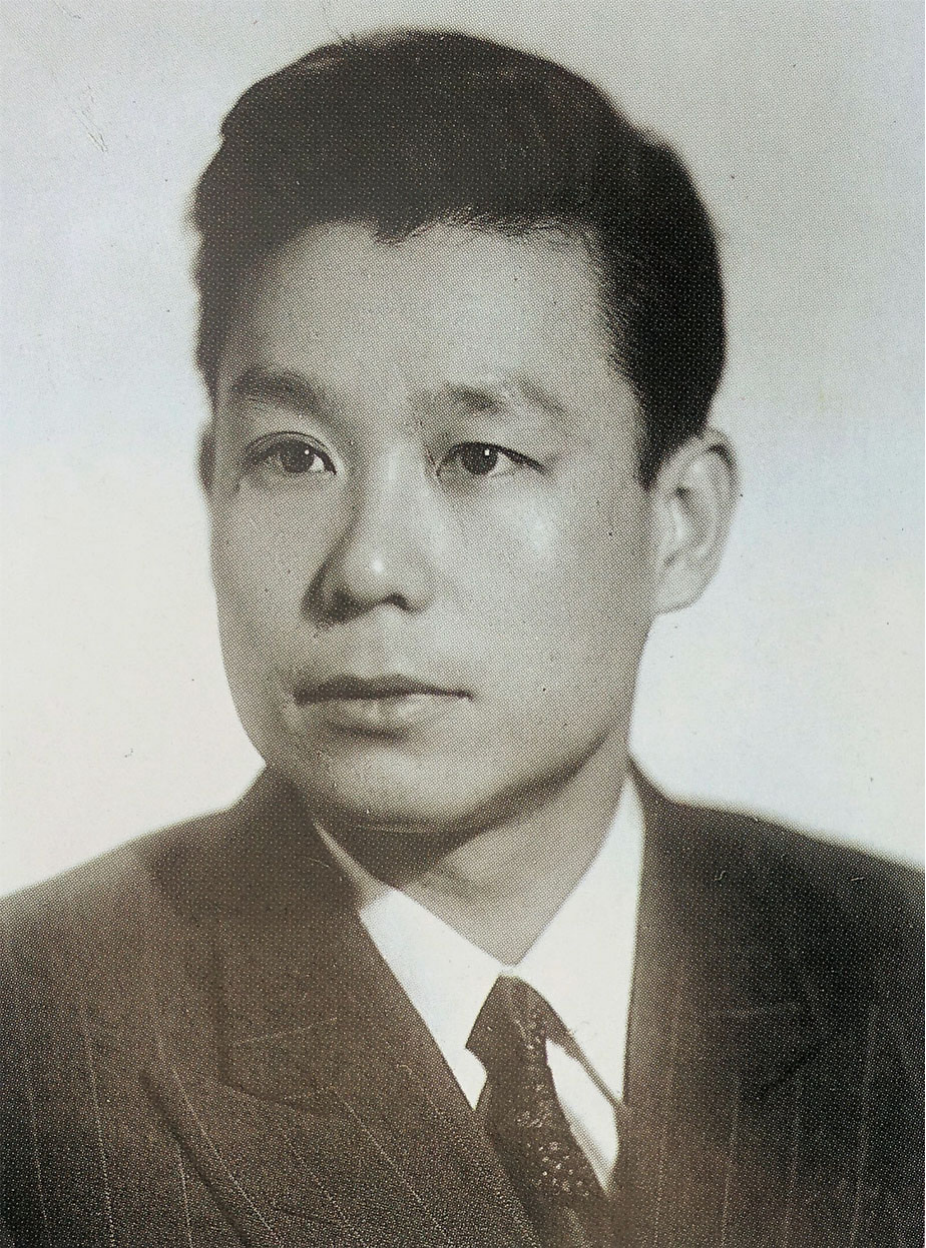
**Lian-Cang Xu in Pedagogical Science Academy of RSFSR in 1962**.

After returning from USSR, Dr. Lian-Cang Xu continued to work on the application of information theory in psychology. Based on these work, he discovered the differences of information transmission function among various control equipment, and the results, which was published on *Science China*, received much attention from Western scholars. Besides that, he focused on the stimulus-response compatibility and the mediating effect of language in human-computer interaction. In the late 1960s, Dr. Xu participated in the development project of Chinese first man-made satellite “Dongfanghong-I”. As a member of the leaders, he and his group members finished the first observation experiment of the animals under the weightlessness condition in China. Furthermore, he revealed the physiological and psychological changes of the experimental animals under such conditions (Yang, 1999).

The psychological science in China was damaged seriously due to the impact of “Great Proletarian Cultural Revolution” (GPCR) in 1970s. To rehabilitate the IPCAS and Chinese Psychological Society (CPS) in a short time, Dr. Lian-Cang Xu tried his best to take a lot of measures with other colleagues. In such difficult environment, he still completed two projects: one was the biological effect of laser, specifically on vision, and the other was the earthquake forecasting through the observation of animals’ reaction. As soon as IPCAS was formally reinstated in 1976, he went back to study management psychology and led a team to carry out a survey on employees’ job and life satisfaction, which was appreciated as “Chinese Gallup Poll” by New York Times.

Since 1979, Dr. Lian-Cang Xu began to lay stress on the action research which centers on the organization development. A tool for measuring the effectiveness of concrete managing policies was developed through his efforts, and its validity was verified in oil, coal, aviation, and railway industries. According to the data obtained by this measuring tool, he discovered the causal relationship between economic performance and human resource management. Moreover, he and his partners conducted studies on leadership behavior, management decision-making, personnel training, and risk awareness, all of which were pioneering in China, and he also integrated the productivity, relations of production and culture to propose a “Tripility Theory”, triggering much attention from academia of China and other countries. Cooperating with other world-famous scientists, such as Misumi Juuji, Hofstede, and Federer, he did a series of cross-cultural researches and devoted to finding out whether Management Theory has cultural specificity and applicability in different countries. All these research findings made significant contribution to the discipline construction of management psychology and promoted the development of management science in China.

In order to cater for the needs of economic system reform and promote the research and application of behavioral science, Dr. Lian-Cang Xu and some other scholars appealed for the establishment of Chinese Behavioral Science Society in 1980s. Meanwhile, he paid more attention to the research of management, such as staff training, work values analysis, and other aspects. Apart from the indigenous study, he began to focus on the research progress in other countries, for example, the PM (Performance Maintain) method from Japan and the Repertory Grid method from U.S. The introduction of these methods to China enhanced the development of industrial psychology research effectively.

Dr. Lian-Cang Xu had abundant works through his life, including more than ten academic monographs, such as *Management Psychology* (1986) with Sheng-Zhong Lu (卢盛忠), *Organizational Management Psychology* (1988) with Wen-Quan Ling (凌文辁), *Research on Leadership Behavior* (1991) with Lin-Lin Yang (杨林林), *Organizational Behavior* (1993), *Management Psychology and Its Application*: *Serve for the Management in Hospital* (1993), and *Management Psychology out of Jungle* (2007), and more than one hundred papers on international and Chinese journals (Liang, 2009). He and his student Kan Shi’s (时勘) research achievements on leadership behavior and *Intelligent Simulation Training* were awarded three prizes for Science and Technology Progress (STP): one second class prize for STP of Chinese Academy of Sciences (CAS), one second class prize for STP of National Light Ministry of China^4^, and one second class prize for STP of National Petroleum Industry Ministry of China^5^. To honor his significant contribution in psychology, CPS conferred the Life Achievement Award in 1999 which is the top prize on him (Fig. 3).

**Figure 3:**
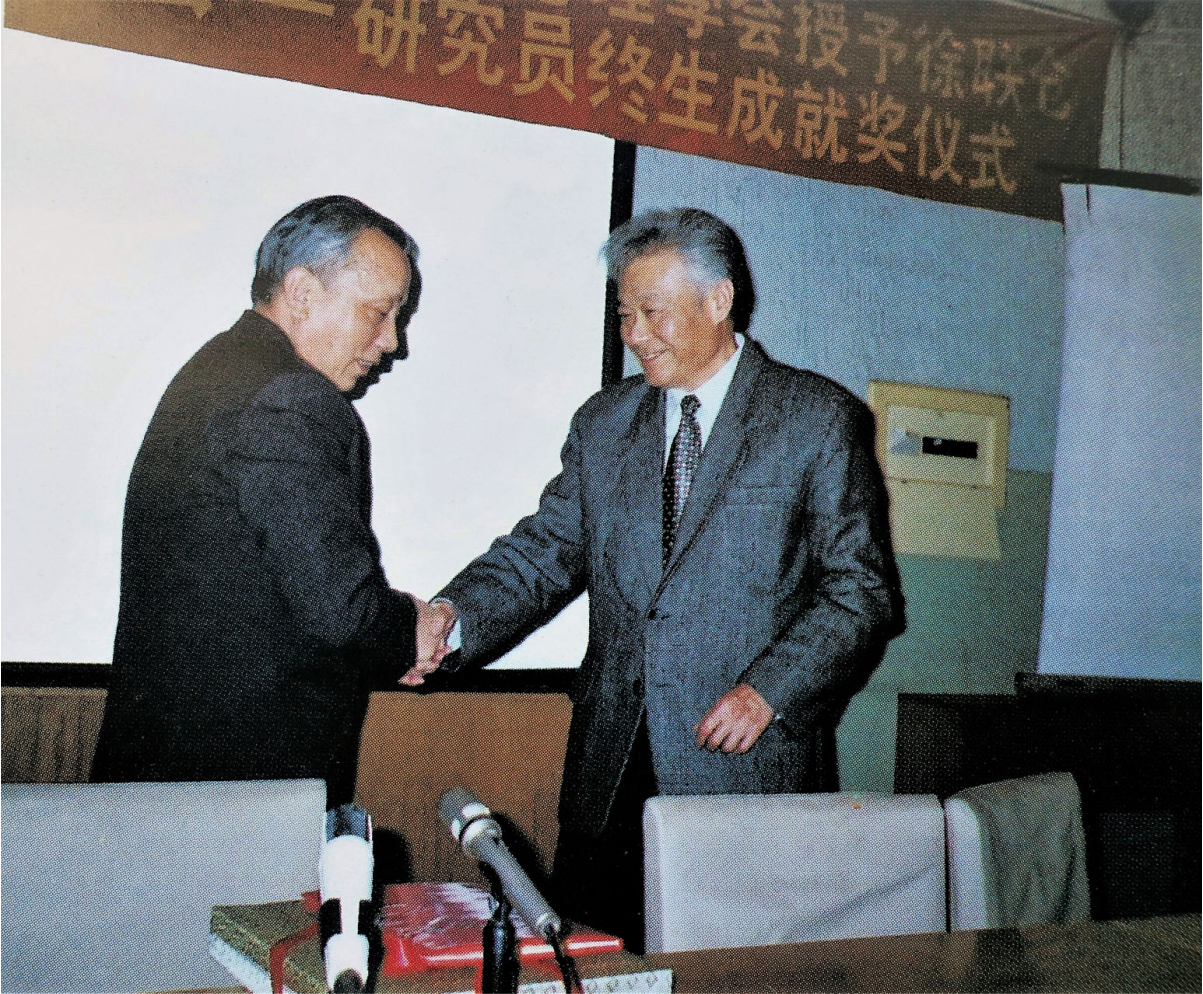
**Dr. Lian-Cang Xu was awarded Life Achievement by Yong-Ming Chen (陈永明), who was then the president of CPS, in 1999**.

 In addition to the research work, Dr. Lian-Cang Xu held many important positions in domestic and International academic institutions. His duties and titles included the director of IPCAS (1983–1987), secretary general of CPS (1978–1985), executive committee member of International Association of Applied Psychology (IAAP, 1984–1998), vice-president of Chinese Behavioral Science Society (1985–1988), vice-president of Chinese Association of Social Psychology (1990–1993), and the chief editor of *Acta Psychologica Sinica*, etc (Jin, 1996).

As a model in Chinese psychological circle, Dr. Lian-Cang Xu studied rigorously, explored diligently, and dared to innovate. For the development of Chinese psychology, he was strongly willing to be dedicated to his work, and trained a group of psychology doctorate and master for China. His conscientious working attitude and generous character were deeply respected all over the world.

## Footnotes

1 Now it is called Philosophy College.

2 The institution was renamed General and Educational Psychology Research Institute of the Union of Soviet Socialist Republics (USSR) Pedagogical Science Academy in 1970, and has been returned to its original name “L.G. Schukina’s Psychological Institute” since 1992.

3 The Kandidat Nauk degree is equal to the doctor’s degree in the States.

4 It has been integrated into Ministry of Industry and Information Technology of China.

5 It has been integrated into National Energy Administration of China.
